# Brand Potential User Identification Algorithm Based on Sentiment Analysis

**DOI:** 10.3389/fpsyg.2022.906928

**Published:** 2022-05-30

**Authors:** Hongxia Li

**Affiliations:** School of Arts, Shandong Management University, Jinan, China

**Keywords:** sentiment analysis, brand potential users, identification algorithm, potential customer identification, sentiment dictionaries

## Abstract

This paper firstly compares the current research status of text sentiment analysis and potential customer identification, and introduces the process of building sentiment dictionaries and feature selection, feature screening, and common classification algorithms in text analysis. Secondly, around the most used tool for sentiment analysis, sentiment dictionary, the sentiment polarity discriminative rules of sentiment words are studied. In response to the shortcomings of using a single recognition algorithm in the current process of building sentiment dictionaries, an improved integration rule is designed and an automatic construction method for domain sentiment dictionaries in the social media environment is proposed. Then, this paper analyzes the sentiment topic information existing in user-generated content and adds the domain sentiment lexicon to the joint sentiment topic model as *a posteriori* information to extract the sentiment topic features, based on which the feature engineering study of potential customer identification is conducted and the feature set is constructed. In addition, a sample resampling method and a diverse integration framework for unbalanced data are designed to work together for the prospect identification task under data skewing in response to the category imbalance in real data. Finally, an experimental study is conducted using a social media text corpus to validate the proposed method in this paper. The proposed domain sentiment lexicon construction method and the joint domain sentiment topic-based lead identification method show good performance in different control group experiments. This paper provides an in-depth study on the construction of domain sentiment lexicon and imbalance classification in theory and provides solutions for companies to discover potential customers in practice, which has certain theoretical significance and practical value.

## Introduction

Potential customers are an uncertain category of target groups that enterprises seek in the competitive market and can create growth in market share, product sales, and other related business indicators for enterprises in the future period, which is an important guarantee for their potential profit. If an enterprise can identify customers with potential purchase intention at an early stage and carry out precise marketing to potential target users, it can minimize transaction costs and maximize transaction probability, thus saving a lot of marketing and operation costs and increasing profitability, making the enterprise more competitive and stand out in the fierce business competition. Therefore, potential customers are very important to promote the long-term development of enterprises, and the identification of potential customers is an important task for enterprises. In the context of the big data era and the booming development of e-commerce platforms, users who buy furniture on e-commerce platforms will post online shopping reviews on the platform, and the reviews include product attributes, services, coordination, and other aspects, which all imply the user’s demand for product improvement. The e-commerce platform has become an important venue for user feedback, bringing together many online shopping reviews. If furniture e-commerce companies can quickly grasp the user needs in online shopping reviews, they can improve existing products and services promptly and find the future direction of product development and inspiration for rapid iteration, thus firmly grasping user stickiness and improving core competitiveness.

However, user-generated content is characterized by multi-source heterogeneity, diverse lexical syntax, and constantly changing sentiment with the topic, which cannot solve the problem of potential customer identification according to general text classification techniques. The domain specificity of sentiment is the first problem that needs to be solved. Emotion is a domain-specific product with obvious domain characteristics. For example, when describing the start of a car, it is generally believed that the faster the better, i.e., in the field of cars, “fast” is a positive emotion. It is noted that the words under some themes are still not easy to summarize the meaning of the theme, so this article discards some of them. In the end, this article selects six themes from the extracted themes: noise, power, fuel consumption, 4S shop service, price, and space. However, when describing the accuracy of a watch, if it is fast, it means that it is not accurate, i.e., in the domain of watches, “fast” is a negative emotion. Therefore, domain-focused sentiment analysis is the only way to truly reflect the emotional color of the object and has more practical value. The main tool for sentiment analysis is sentiment dictionaries, but most of the current sentiment dictionaries are not well adapted to domain dependency and cannot cover new words in social media in time and accurately identify the sentiment tendency of unlisted candidates, so there is a need to study the method of building sentiment dictionaries in specific domains. Besides, in the actual prospect identification task scenario, feature engineering needs to be carried out based on the business characteristics of different domains to construct suitable feature sets, which is content worthy of in-depth study. Meanwhile, in the real environment, the target potential customer group accounts for a low proportion of the overall users, and this sample sparsity problem is called sample imbalance in the classification problem, and the study of how to perform potential customer identification under data skew is also a problem that needs to be considered.

In terms of practical application value, the benefits of low-cost, high-efficiency identification of users with potential purchase intentions for enterprises are not only to achieve good customer relationship management, provide differentiated services for different customers, improve the quality of enterprise services, and enhance the core competitiveness of enterprises, but also to carry out market segmentation to take accurate marketing, while reducing inventory squeeze, greatly reducing the blindness of marketing brought. The cost of marketing blindness is greatly reduced, and the competitive advantage of the product is expanded for the enterprise, bringing sustainable profit growth. On the other hand, sentiment analysis technology can also provide an in-depth exploration path for enterprises to monitor product word-of-mouth, discover product quality defects, guide product sentiment communication, and enable enterprises to provide more satisfying and high-quality services to users. In this paper, we focus on sentiment analysis in the automotive domain, using user-generated content in the automotive domain in the social media environment as a data source to build a sentiment lexicon in the automotive domain on the one hand and apply the domain sentiment lexicon to a potential customer identification task under an unbalanced data set, on the other hand, to design a potential customer identification method with joint domain sentiment themes to discover users with potential car purchase intention.

## Related Works

The visualization of multi-level information includes two types of visualization methods: time combined with other information and other visualization methods based on multi-level information. Time is an indispensable attribute for timely texts such as news and blogs, and visualization of temporal information is widely proposed based on this situation. The most direct and effective way is to sort the information from left to right according to the timeline. CBLAS is a user-centered platform that supports assessment practice for clinical faculty and learning for residents, as well as administrative work for staff based on user, needs assessment, and the operationalization features of CBME assessment (Chakraborty et al., 2020). Or the shared experience of users who have purchased a car, or who have not yet purchased but are not potential customers. This situation is called data skew in classification tasks, and the dataset in this case is an imbalanced dataset. With this system, clinical faculty have a more proactive approach to milestone assessment activities, and EPAs and learners can schedule their learning to improve their effectiveness. Combining both text mining techniques and perceptual engineering, a classification tree was constructed for effective market segmentation, and correspondence analysis was used to capture users’ perceptions of affective features to identify the core characteristics that best describe different segments. Using theories related to perceptual engineering, a framework is built to help industry practitioners translate different customer needs into attractive alternatives while maintaining a manageable manufacturing cost (Vermeer et al., 2019). A combined text mining and perceptual engineering model are constructed that extracts key perceptual descriptive terms based on actual customer reviews to predict consumer preferences for product design while reducing some of the repetitive tasks of designers.

The mining and analysis of product reviews are of great importance for both business development and consumer choice, and fine-grained review mining of product features has become a hot topic of research. It is believed that the goal of review mining is to determine the relationship between opinion holders, topics, statements, and sentiments. In practical applications, a review sentence often contains multiple features of a product, and users may hold different opinions about different product features. It is more relevant to mine the sentiment of product features (Kaur and Kautish, 2019). Feature-level sentiment analysis extracts opinions expressed on different features of different entities and determines users’ attitudes toward the features by studying the features and the modifiers corresponding to the features. Then, the sentiment vocabulary is analyzed to determine its polarity. PreLM-FT fine-grained sentiment analysis is used to transform user comment data into user preference scores in a context where only a small amount of label data is available (Xia et al., 2020). A semi-supervised self-training-based feature extraction method is proposed to find similar words of feature seed words on the unlabeled dataset by word vector model to build the set of feature words most relevant to the dataset for each feature. Nowadays, feature extraction using semi-supervised learning is becoming increasingly widespread, which combines the advantages of supervised learning and unsupervised learning and requires only a small amount of manual labeling to extract commodity features more accurately.

In this paper, we take the product reviews of the cell phone industry as an example for an in-depth study and build a review mining system based on sentiment analysis. Reviews on shopping sites are usually simply divided into positive and negative reviews, which are evaluations of the product. However, the good and bad of a product cannot be covered by points, and fine-grained sentiment analysis research should be conducted. The goal of this paper is to extract the key attributes of the product and score the sentiment evaluation of these attribute information to establish a sentiment analysis model, which in turn facilitates the targeted improvement of the product’s shortcomings. Data feature extraction and dimensionality reduction are made to improve the commodity feature extraction method, and a feature extraction method based on word frequency and semantics is proposed. The feature word set is constructed by high-frequency nouns and association rules, word2vec is used for word vector training, and then, the feature word set is expanded by calculating the cosine similarity between vectors, and finally, a complete dictionary of commodity features is obtained after manual classification. The experiments show that the performance of this method is superior in terms of accuracy, recall, and F1 value.

## User Identification Algorithm Design for Sentiment Analysis

Sentiment dictionary is an effective technical tool for sentiment analysis. With the diversified development of social media content, the domain characteristics of sentiment dictionaries are becoming increasingly obvious, and new words and proper nouns are constantly emerging (Bilro et al., 2019). Traditional sentiment dictionaries are not well adapted to domain dependency, timely coverage of new words in social media networks, and accurate identification of sentiment tendencies of unregistered candidates, and the method of building sentiment dictionaries based on automatic expansion within the domain of seed words has become a popular research content at present.

Project Management Institute (PMI) is a statistic that uses the degree of textual co-occurrence between two words or phrases to measure the distance between them, so PMI is used to calculate the correlation between candidate words and seed words. Assuming that $w_{1},w_{2}$ are used to represent two words or phrases, respectively, the PMI is calculated as


(1) $$PMI\left(w_{1},w_{2}\right)=\ln\frac{p\left(w_{1},w_{2}\right)}{p{\left(w_{1}\right)}^{2}p{\left(w_{2}\right)}^{2}}$$

Equation (1), $p\left(w_{1}\right)$ denotes the probability of $w_{1}$ occurring in the corpus, denotes the probability of simultaneous occurrence in the corpus, and $PMI\left(w_{1},w_{2}\right)$ denotes the $w_{1},w_{2}$ degree of association (Khalid et al., 2020). The PMI formula calculates the average association degree of candidate words with all positive and negative seeds in the seed lexicon, which is used as the sentiment score of candidate words, and the formula is as follows.


(2) $$\begin{array}{l} Score\left(word\right)=\frac{1}{N_{pos}}\overset{N_{pos}}{\underset{i=1}{\sum }}PMI\left(word,posSeed_{i}\right)\\ \;\;\;\;\;\;\;\;\;\;\;\;\;\;\;\;\;\;\;\;\;\;\;\;\;\;\;\;\;\;\;\;\;\;+\frac{1}{N_{pos}}\overset{N_{pos}}{\underset{j=1}{\sum }}PMI\left(word,posSeed_{j}\right) \end{array}$$

The Hierarchical Category Sentiment Analysis (HCSA) algorithm can summarize the main difficulties or points that need to be addressed when processing them into three parts. First, because the structure of sentiment categories in the Hierarchical Category Sentiment Analysis task is designed to be different from the mainstream sentiment analysis methods that are tiled, i.e., all sentiment categories are treated equally, but instead, different shallow sentiment categories are deeply layered and dispersed (Lutfi et al., 2018). Similarly, in the identification of potential customers, we also pay more attention to the identification accuracy of minority potential customers. Therefore, this section proposes a potential customer identification method for imbalanced data from the perspectives of sample resampling and model ensemble combination T.

**Figure 1:**
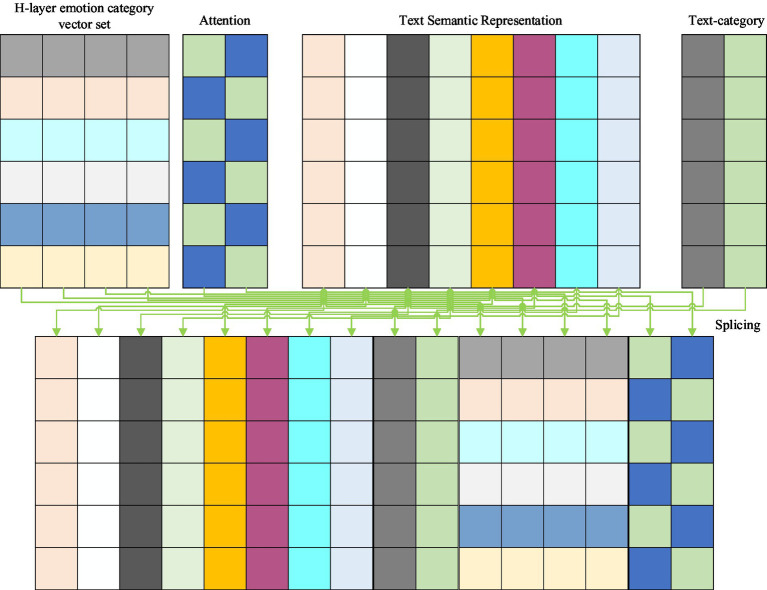
Schematic diagram of the text-emotion category attention layer.

Figure 1 shows the interaction between the attention layer of the sentiment text information and the current h-th level sentiment category information in the sentiment feature acquisition unit. Where V is the text-semantic representation obtained from the sentiment text input unit, for the sentiment opinion category, all sentiment categories in each layer are represented by random initialization vectors in this algorithm model, and Ch represents the spliced set of vector representations of all sentiment opinion categories under the current h-th layer. Then, the text-semantic and sentiment category vectors are computed for text-emotion category attention.

The main idea of the category-text attention mechanism designed and implemented in the attention layer of the hierarchical messaging unit is to use attention computation at the current h-th level to capture the semantic contribution of text characters to different sentiment categories at this level, e.g., the four characters “food” and “delicious,” for example, the four characters “food” and “delicious” in the text have a higher semantic contribution to the sentiment category “Food.” That is, the idea of category-text attention is to focus text characters on different emotional opinion categories, while the idea of text-emotion category attention is to focus emotional opinion categories on different text characters.


(3) $$D^{h}=V⊗S^{h-1}$$

(4) $$A_{c}^{h}=soft\max\left(C^{h}\cdot \coth(W_{D}^{h})\cdot {\left(D^{h}\right)}^{T}\right)$$

(5) $$v_{c}^{h}=mean\left(A_{c}^{h}\cdot D^{h}\right)$$

The role of the dependency information transfer layer within the hierarchical information transfer unit is to combine the sentiment semantic information from the h-th level of this layer and the analysis results output from the hierarchical sentiment classification unit of this layer to construct dependency information and pass it to the next h + 1-th level for processing (Alaei et al., 2019).

For constructing the integration information for passing to the next level h + 1, it is necessary to refer to and assimilate the sentiment classification prediction results of the current level h. This is to fit the inclusion relationship or parent–child category dependency relationship between the sentiment categories in two adjacent levels in the proposed hierarchical category sentiment analysis task. The hierarchical sentiment category vector representation dimension is 256. In the emotional feature acquisition unit, there are three types of convolutional neural network window sizes, which are 2-, 3-, and 5-character lengths, and the number of convolution kernels for each size is set to 100.

The Word2vec technique can map each word in the corpus into a vector of fixed dimensions, and when the word vector constructed using this method is used to calculate the pinched cosine distance between two words, the pinched cosine value of the vector reflects the semantic similarity between the two words. The average semantic similarity between the candidate word and all positive and negative seeds in the seed lexicon is calculated by the entropic cosine formula to derive the sentiment score of the candidate word, and the related formula is as follows:


(6) $$\sin\left(w_{1},w_{2}\right)=\frac{\overset{n}{\underset{i=1}{\sum }}x_{i}^{2}y_{i}^{2}}{\sqrt{\overset{n}{\underset{i=1}{\sum }}x_{i}^{2}}-\sqrt{\overset{n}{\underset{i=1}{\sum }}y_{i}^{2}}}$$

Like the use of PMI to determine the sentiment polarity of candidate words, when the threshold H2 is larger, the number of sentiment words identified is smaller; when the threshold H2 is smaller, the number of sentiment words identified is larger, but more irrelevant sentiment words are also identified, which results in the degradation of sentiment lexicon performance (Li et al., 2019). Integrated learning is a common and effective machine learning method, which is based on the idea of first training a set of base learners and then designing specific rules to fuse the results of the base learners as the final model output. Integrated learning is a framework in which various basic machine learning methods can be embedded, for example, a common base classifier in random forests is a decision tree.

**Figure 2:**
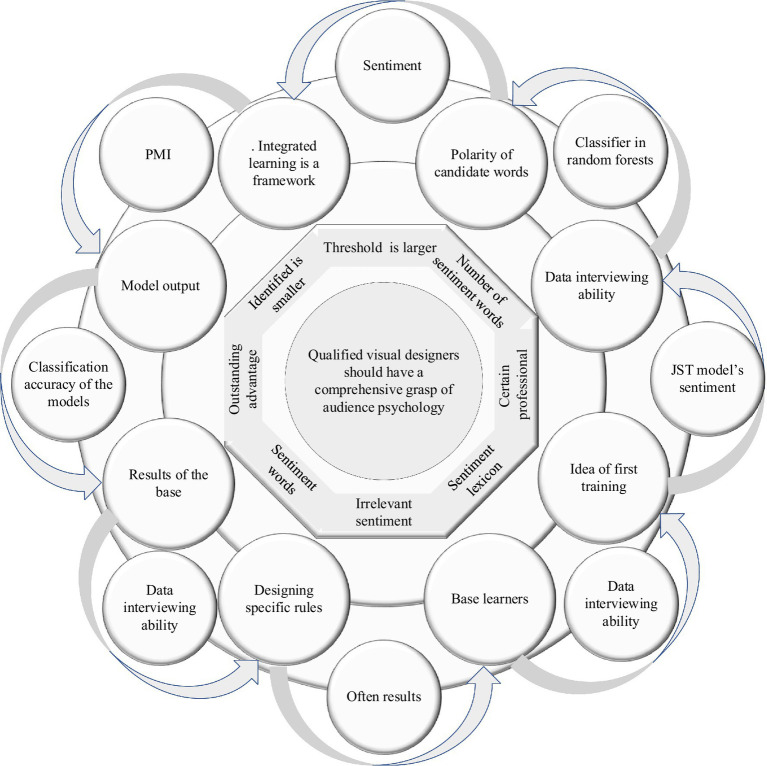
Structure of Japan Science and Technology (JST) model with the introduction of prior knowledge.

In Japan Science and Technology (JST) model’s sentiment belongs to a special topic type, and the direct use of JST models often results in poor classification accuracy of the models due to the lack of *a priori* knowledge, and the effect of their extracted sentiment topic features is difficult to achieve better results. Therefore, it is necessary to incorporate prior knowledge into the JST model (Polyakova et al., 2019). The F1-measures are 69.96%, 72.87%, 74.21%, and 75.75%, respectively. The F1-measures of the HCSA (without hierarchy) and HCSA algorithms proposed in this paper are 75.49% and 77.37%, respectively. The domain sentiment lexicon introduced in this section is used as prior knowledge and retrained to construct the JST model. The structure of the JST model after the introduction of prior knowledge is shown in Figure 2.

The V in the model structure denotes the set of all words in the whole corpus, i.e., the set of words, and the hyperparameter denotes the transfer matrix of prior information. Since the domain sentiment dictionary contains sentiment labels (commonly, i.e., positive and negative labels) for each sentiment word, if the domain sentiment dictionary contains the current word w and the sentiment label corresponding to w, then assign the current word w and the sentiment label l to 1 and the other components to 0. The process is calculated as follows:


(7) $$\lambda_{lw}=\left\{\begin{array}{c} 1,\;\;\;\;\;\mathrm{if}\;S\left(w\right)=0,\\ 0,\;\;\;\;\mathrm{otherwise}\;\;\; \end{array}\right.$$

**Figure 3:**
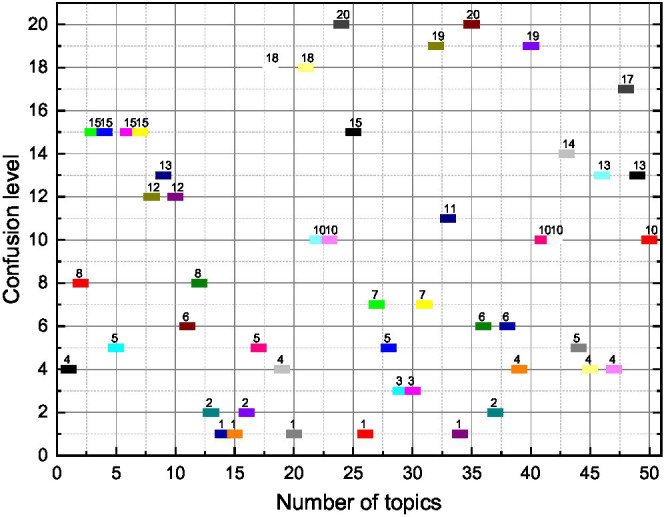
Trend of the number of topics on the confusion level.

Theoretically, if the number of topics is reasonable and the iterations converge, it is possible to summarize the meaning of the topics by the words with higher probability distribution in each topic, and thus, the sentiment topic extraction is more desirable (Rupapara et al., 2021). According to the perplexity of the training set on a different number of topics, there is a clear inflection point when *K* = 9. Therefore, observe the distribution of words when the number of topic labels is 9. It is noted that the words under some topics are still not easy to summarize the meaning of the topics, so some of them are discarded in this paper. Finally, six topics are selected from the extracted topics: noise, power, fuel consumption, 4S store service, price, and space. This is because when the value of H increases gradually, it can be known from Equation (6) that the number of emotional words added to the emotional dictionary will gradually decrease. However, when the value of H is small, too many emotional words are added to the emotional dictionary, which inevitably introduces noise, so the accuracy rate is reduced. These six topics can be summarized in a better way, and the interpretability is better. According to the probability of the distribution of these six emotional topics under different emotions, a total of 12 features under positive emotion distribution and negative emotion distribution were converted into the emotional features of the topics, and the probability values of the topic distribution under positive and negative emotions were used to represent the feature values, as shown in Figure 3.

In the real prospect identification task scenario, there are few users with real potential car purchase intention, which is shown by the fact that most of the review texts are meaningless noise texts, or the shared experiences of users who have already purchased cars, or have not yet purchased but are not potential customers either. This situation is called data skewing in the classification task, and the dataset in this case is unbalanced. In the case of unbalanced data, the performance of traditional machine learning methods can be significantly degraded, inapplicable, or even have an inflated accuracy rate (Valle-Cruz et al., 2022). For example, in a fraud detection task scenario, the sample space contains 100 samples, where the ratio of minority class to majority class is 1:99. If the original machine learning methods such as SVM are used directly, the accuracy is as high as 99% even if the minority class is predicted incorrectly, but it is obviously meaningless because the minority class of concern is partially wrong, and similarly the minority class of potential customers is also more concerned in the identification of potential customers (Aswani et al., 2018). In the era of big data and the booming development of e-commerce platforms, users will post online shopping reviews on the e-commerce platform after purchasing furniture. The content of the reviews includes product attributes, services, coordination, etc. The accuracy of customer identification is also more concerned with lead identification. Therefore, this section proposes a prospect identification method for unbalanced data from the perspective of both sample resampling and model integration combination.

Boosting is an integrated approach to improve the overall classification performance by reducing bias. It adopts the idea of incremental training, where each training is performed by relying on the residuals of the last prediction result and the real label value as the new label, so the base classifier is often a regression tree, which represents the boosting tree model (Lou et al., 2019). To solve the actual situation that there are fewer potential customers and more non-potential customers in the dataset in the process of potential customer identification, this paper proposes a new classification framework for unbalanced datasets.

## Experimental Design for Brand Potential User Identification

Combined with the automatic domain sentiment dictionary expansion framework in the social media environment in “User Identification Algorithm Design for Sentiment Analysis” section, the domain sentiment dictionary is first constructed in the existing corpus according to the framework method, and the text sentiment binary classification is performed on the test set based on this sentiment dictionary, while the sentiment dictionaries constructed by other methods are subjected to text sentiment binary classification experiments on the test set, and finally, the classification accuracy differences of different sentiment dictionaries are compared to verify. Finally, we compare the accuracy of different sentiment dictionaries to validate the proposed method of automatic domain sentiment dictionary expansion framework in the social media environment (Leicht-Deobald et al., 2019). Accuracy is chosen as the evaluation metric here.

**Figure 4:**
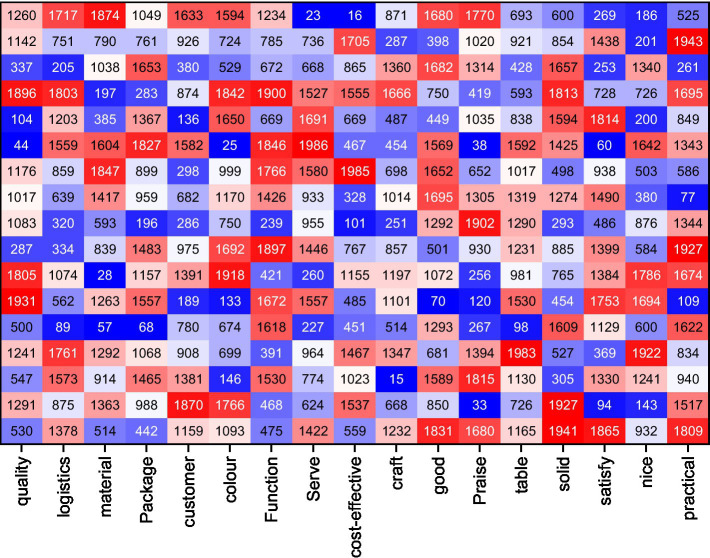
The co-word matrix.

When using sentiment dictionaries for text sentiment classification, this paper uses a linear weighting method to calculate the sentiment score of texts, and this method assumes that the sentiment intensity is linearly superimposable. At the same time, all sentiment words in the sentiment dictionary are given fixed weights, and in this paper, the weight of positive sentiment words is set to unit 1, and the weight of negative sentiment words is set to unit −1 (Wang et al., 2019). First, the text is scanned from front to back afterword separation, and if the current word matches a positive sentiment word in the sentiment dictionary, a forward weight of 1 is added, and if it matches a negative word, a forward weight of −1 is added. Then, the encountered Negative words and degree adverbs are logically judged: among the words in the specified context window (the window size is taken to be 4 in this paper), the occurrence of negative words leads to opposite weights and the occurrence of degree adverbs doubles the weights, and finally, the sentiment of the sentence is judged based on the positivity or negativity of the total weighted score of the text, as shown in Figure 4.

Network, as a graph theory and complex network modeling tool, is based on the Python language and is internally configured with standard graph and complex network analysis algorithms, capable of simulation modeling and complex network data analysis. It can improve existing products and services in time, find the future development direction of products, and find inspiration for rapid iteration, to firmly grasp user stickiness and improve core competitiveness. In addition, Networks include common graph theory algorithms, nodes can be set to arbitrary data, and edge dimensions can be adjusted at will, making it powerful and easy to use (Yue et al., 2019). With the help of Networks, it is possible to analyze the structure of networks, build models of networks, design new network algorithms, and drawing networks. In terms of storage, it is possible to store both standardized and non-standardized data formats. In Networks, the basic approach to create a semantic network graph is as follows: first, create an empty network graph; second add nodes and edges, where the size of the points and the width of the edges are adjustable; and finally generate a graph book to save locally.

**Figure 5:**
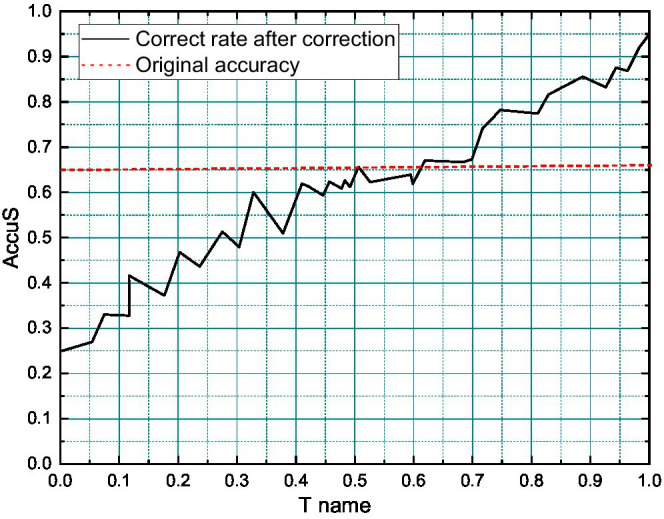
Variation of single-sentence-level correctness with *τ* values.

In the current research scenario, we can consider a tweet/tweet as a single sentence, which can be used in the above convolutional algorithm for text analysis (Cambria et al., 2019). To introduce the user’s role features into this algorithm, we tried different combinations, such as: inserting the user’s role feature vector as a component in the sentence into the sentence matrix representation, etc. Pre-experiments show that the best way to join is to splice a user role feature vector on each word representation, as shown in Figure 5.

The main core parameters of this algorithm model are configured as follows: the overall layer category sentiment analysis model layer is set to 4 layers (Barbado et al., 2019). In the sentiment text input unit, the maximum length of the input text is set to 512, the semantic representation dimension is 768, and the dimension of the hierarchical sentiment category vector representation is 256. In the transmission unit, the sentiment category-text attention mechanism dimension is 256. In the cascading sentiment classification unit and the global sentiment classification unit, the nonlinear fully connected neural network dimension is 384. Finally result output unit, the global analysis result share α is set to 0.5 in the cascading dependent prediction method, and the result additive weight of the shallow level parent sentiment category to the deepest level target subcategory is 0.15.

## Results Analysis

### Analysis of the Performance Results of the Sentiment Recognition Algorithm

**Figure 6:**
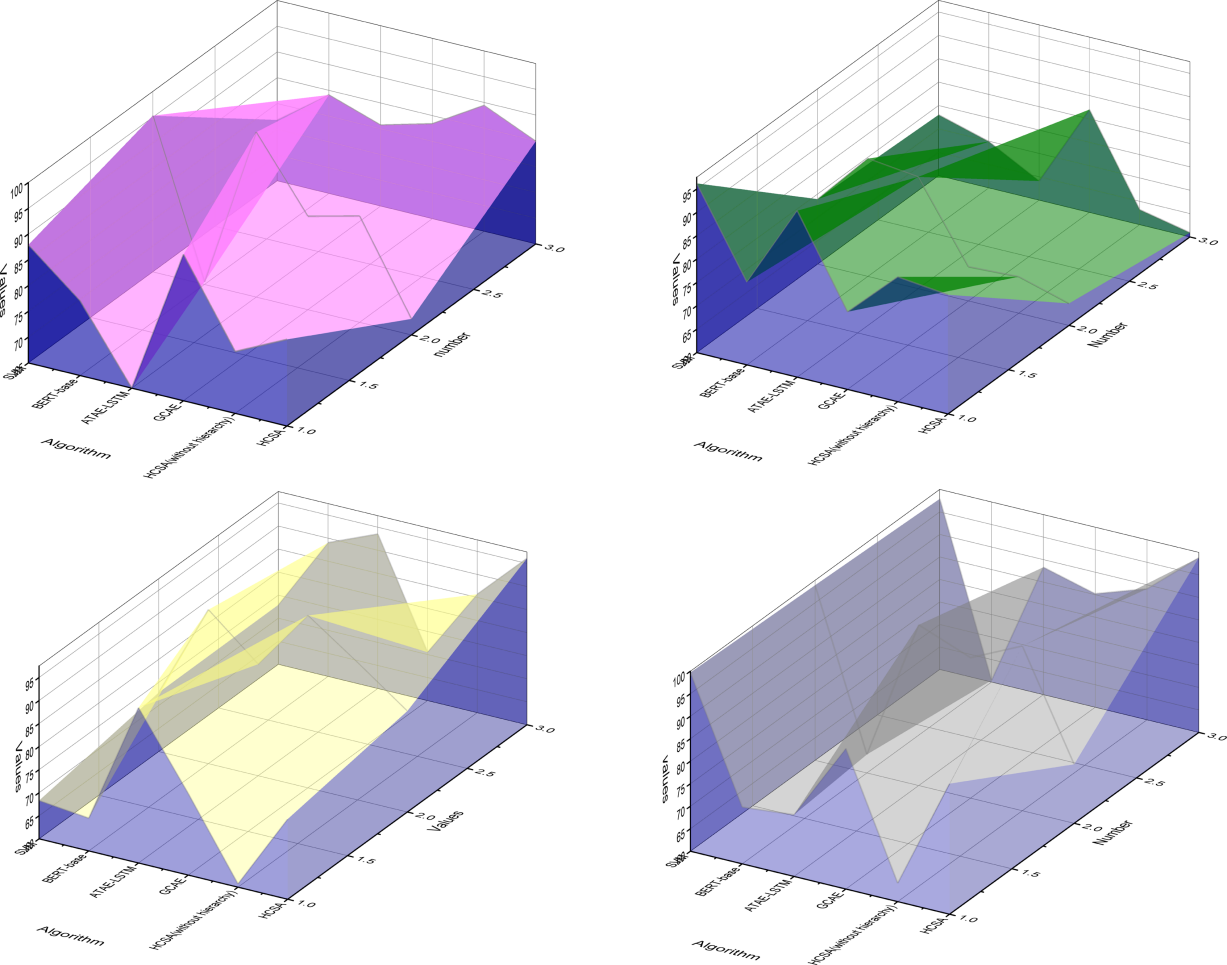
Evaluation index results of each algorithm on the analyzed dataset.

The HCSA (without hierarchy) model is a version of the HCSA model that removes the hierarchical information transfer unit and the hierarchical sentiment categorization unit that links the hierarchical information. The HCSA (without hierarchy) model version refers to the HSCA algorithm model after removing the hierarchical information transfer unit and the hierarchical sentiment classification unit, which are the model structures linking the hierarchical information, and this model can be regarded as having the same task processing perspective as the SVM, BERT-base, ATAE-LSTM, and GCAE algorithms used in the above comparison, i.e., ignoring the multi-layered characteristics of sentiment categories and view all layered sentiment categories equally, which is convenient for comparative analysis. The HCSA model is the complete algorithmic model proposed in this paper, which includes the independent processing of different hierarchical sentiment categories and the information interaction between the hierarchical levels. “Fast” is a negative emotion. Therefore, the emotional analysis focusing on the field can truly reflect the emotional color of objective things, and it has more practical value. The metrics of the above six algorithmic solutions on the sentiment analysis dataset of this paper are shown in Figure 6.

From Figure 6, under the sentiment analysis dataset, the F1-measure of the composite metrics of the comparison algorithms SVM, BERT-base, ATAE-LSTM, and GCAE are 69.96%, 72.87%, 74.21%, and 75.75%, respectively, and the HCSA (without hierarchy) and HCSA algorithms proposed in this paper The F1 indexes of HCSA (without hierarchy) and HCSA algorithm proposed in this paper are 75.49% and 77.37%, respectively. The F1 index of the HCSA algorithm, i.e., HCSA (without hierarchy) algorithm, which removes the part of the hierarchical information transfer unit and hierarchical sentiment classification unit, is slightly lower than that of the GCAE algorithm with suboptimal performance in comparison by 0.26%, but the full version of the HCSA algorithm in this paper is higher than that of the GCAE algorithm with a suboptimal performance by 1.62%. It indicates the effectiveness of the hierarchical information transfer unit and the hierarchical sentiment classification unit in the HCSA algorithm model of this paper, and its processing of information transfer and interaction among different layers, etc., better fits the hierarchical characteristics of the hierarchical sentiment analysis task itself and improves the performance of the whole model on the hierarchical sentiment analysis task.

**Figure 7:**
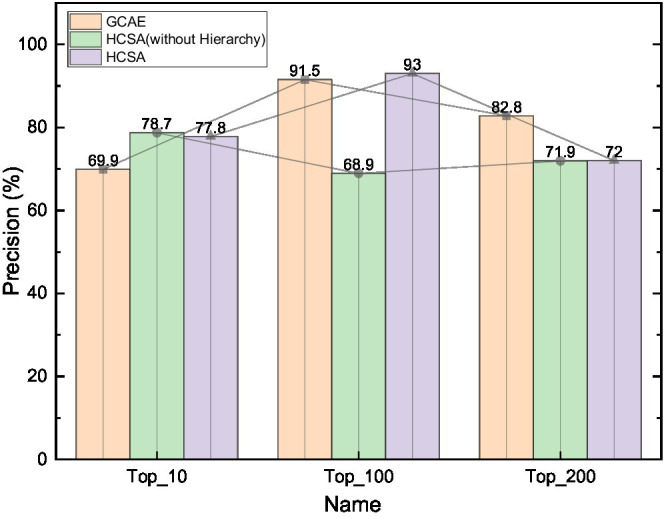
Variation curve of precision with category complexity.

However, it can be seen from Figure 7 that the performance of various sentiment analysis algorithms, including the best-performing HCSA algorithm, does not exceed 80% of the F1-measure in the performance of the proposed hierarchical sentiment analysis task, which is inferior to the performance of these mainstream algorithms in common sentiment analysis tasks. This is something worthy of further study. At the same time, in the real environment, the target potential customer group accounts for a low proportion of the overall users. This kind of sample sparse problem is called sample imbalance in the classification problem. It is also necessary to consider how to identify potential customers under data skew. This is mainly because the new hierarchical sentiment analysis task proposed in this paper adds a new dimension of hierarchical refinement of sentiment categories compared with the mainstream sentiment analysis task, and the sentiment categories in the dataset are therefore more complex and numerous, making it more difficult for the algorithm model to analyze them.

The variation curves of Precision and Recall metrics of GCAE, without hierarchy (HCSA) and HCSA algorithms with the best overall performance after retaining specific hierarchical sentiment categories in the dataset are shown. The horizontal coordinates on the line graph from left to right are the criteria of the dataset after retaining the frequency of Top10, Top100, Top200, and all 304 hierarchical sentiment categories, from left to right, the complexity of the sentiment categories contained in the dataset of public opinion analysis gradually increases and the problem of small samples becomes more serious.

### Analysis of Experimental Results

**Figure 8:**
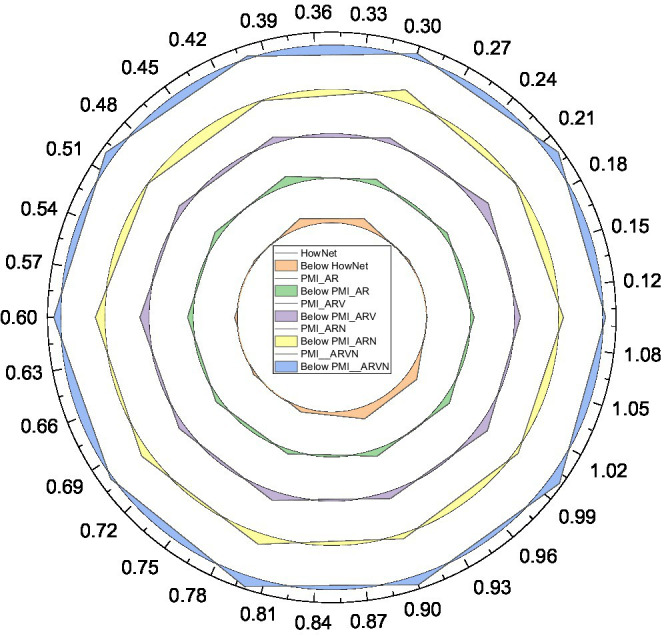
Classification accuracy of Project Management Institute (PMI)-based sentiment dictionary for different H1 values.

Firstly, we determine the optimal threshold H1 value for expanding the sentiment dictionary using the PMI method and calculate the classification accuracy of the sentiment dictionary generated based on PMI in each case at this time by taking H1 in intervals of 0.1 from 0 to 1. The results are shown in Figure 8.

From the longitudinal analysis in Figure 8, the classification accuracy of sentiment dictionaries generated from all candidate word sets tends to increase first and then decrease when the threshold H increases from 0.1 to 0.3 and then to 1.0. This is because when the value of H increases gradually, the number of sentiment words added to the sentiment dictionary decreases gradually at this time, as shown by Equation (6). However, when the value of H1 is small, too many sentiment words are added to the sentiment dictionary, and noise is inevitably introduced, so the accuracy decreases. Instead, different shallow emotional categories are deeply layered and diffused. Doing so brings the benefits of further refinement from the sentiment analysis perspective, but also makes the multi-level sentiment categories more complex, the deeper the sentiment categories are. At the same time, when H takes a larger value, although the number of sentiment words added is controlled and the possibility of introducing noise is reduced, the number of sentiment words recognized decreases, making the cover of the sentiment dictionary incomplete and causing the accuracy rate to decrease. Therefore, there is a critical point where the sentiment dictionary has a certain number of sentiment words to ensure the coverage, and the proportion of noise is controlled to a minimum, and the classification accuracy of the sentiment dictionary reaches the highest at this time.

From the cross-sectional view of Figure 8, the different lexicons in the sentiment dictionary do have significant differences in the classification effect of the sentiment dictionary. With a fixed threshold H, the sentiment dictionary constructed when the candidate word set, i.e., PMI_ARVN, has better classification accuracy than the sentiment dictionary constructed when the candidate word set is other combinations of an adjective, adverb, verb, and noun. In addition, regardless of the combination of candidate words, the sentiment dictionary obtained by extending the generic dictionary outperforms the sentiment dictionary HowNet without candidate word extension, which indicates that the generic sentiment dictionary performance is significantly inferior to the classification performance of the domain dictionary in a specific textual domain context, which is the significance of the construction of the domain sentiment dictionary. Sampling at a frequency higher than twice the highest frequency of the signal is called oversampling. Sampling at less than twice the highest frequency of the signal is called undersampling.

**Figure 9:**
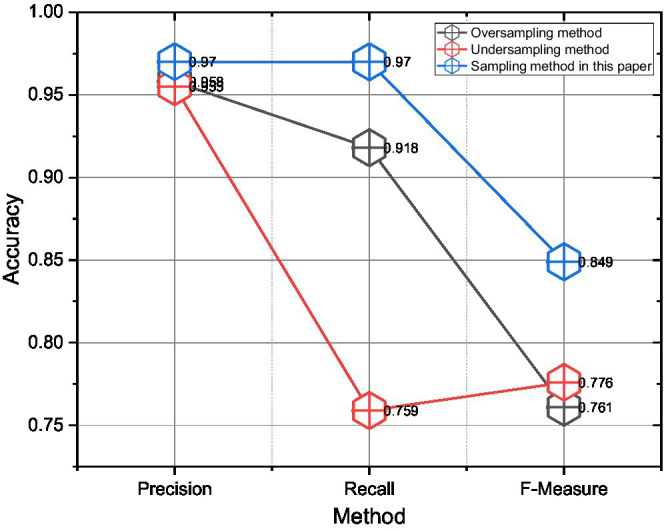
Comparison of the experimental results between the sampling method and the traditional sampling method.

First, when facing the balancing treatment of skewed data, the sample resampling method proposed in this paper and the traditional oversampling and under sampling methods are used to conduct classification experiments on the same unbalanced dataset, respectively, and the results are shown in Figure 9. The traditional under sampling and oversampling are simple and efficient, but the processing of the data is relatively brutal. As can be seen from Figure 9, in the experiments on the dataset of this paper, oversampling a few classes can significantly improve the accuracy and recall rate, while the performance of the undersampling majority class method is average. This is probably because the potential customers in the dataset of this paper are too sparse, so the model can be trained better by using oversampling to enlarge the proportion of minority classes. The sampling method in this paper uses both oversampling and under sampling, and although it only improves the precision rate by 0.4% compared to the oversampling method, it significantly improves the recall rate by 3.5% and the *F*-value by 2.2% in aggregate. The inclusion relationship or parent–child category dependency relationship exists between emotional categories in two adjacent levels. Therefore, using the sampling method in this paper is more effective for subsequent classification in the process of balancing the skewed samples.

Unlike the Boosting integration, the combination structure of the base classifiers is serialized into a chain of classifiers, and the output of the integration depends on the last classifier rather than the overall weighted average. Unlike Bagging, the output of each classifier chain is not directly majority-voted, but rather is stitched together as a feature vector of meta-classifiers and filtered by a meta-classifier again. To validate this integration approach, this paper compares it with both Bagging and Boosting integration frameworks. To control the variables, the data balancing in the preprocessing step is uniformly done by the sample resampling method proposed in this paper, and the base classifier of each integration approach is chosen as a CART classification tree. The AdaBoost method is used here to integrate the CART classification tree as the base classifier.

The integration method in this paper significantly outperforms the corresponding metrics of Bagging by 2.7% and 4.5% in terms of precision and recall, and by 3.8% in terms of *F*-value. Compared to the Boosting integration method, it performs slightly better, with 0.5% and 0.8% improvement in precision and recall, respectively, and 0.7% improvement in F-value. The reason for this is that the integration method in this paper is like the Boosting integration method in that it is composed serially and the latter base classification uses the error of the previous base classifier to adjust the probability distribution of the sample space. However, in the result output, this paper takes the last output of the classifier chain and maps the results of different classifier chains to the feature space of the meta-classifier by stitching, which is a kind of integration method following Stacking. The results show that although the integration approach is more complicated, the metrics such as F-Measure are also improved to some extent, and the important recall metric is the highest among the three, indicating a better check-all rate in identifying potential customers.

## Conclusion

Text sentiment analysis is a common means of analyzing user-generated content in social media settings. In this paper, we chose the perspective of the automotive domain in the social media environment and conducted an in-depth study on the way of constructing domain sentiment lexicon from the perspective of sentiment analysis, and the potential customer identification method of joint domain sentiment topics. The role and application scenarios of domain sentiment information in user-generated content are analyzed, and the sentiment topic feature extraction based on the joint sentiment topic model is investigated for the feature that sentiment information changes along with the topic. To improve the accuracy of sentiment discriminations of sentiment topics, a domain sentiment lexicon is incorporated as *a posteriori* information, and the topic features of the best number of sentiment topics are extracted based on the confusion degree, while a feature set of potential customer identification methods applicable to the field is constructed for the business characteristics of the automotive field in social media. Then, design specific rules to fuse the results of the base learner as the final model output. Ensemble learning is a framework in which various basic machine learning methods can be embedded. For example, the commonly used base classifier in random forest is decision tree. At the model level, to integrate the base classifiers more effectively and diversely, a model integration framework is designed in which the base classifiers are first serially combined into classifier chains, and then, the results of different classifier chains are mapped to a new set of feature spaces, and the outputs are again filtered by the classifiers. A corpus of user-generated content from real social media platforms is collected as experimental data for the experimental study. The comparison experiments show that the domain sentiment lexicon constructed using the domain sentiment lexicon construction method proposed in this paper achieves good accuracy rate results in sentiment classification tasks, while the potential customer identification method designed in this paper for domain sentiment topics still shows good performance in terms of accuracy rate, recall rate, *F*-value, and other metrics under a real sample skewed environment.

## Data Availability Statement

The original contributions presented in the study are included in the article/supplementary material, further inquiries can be directed to the corresponding author.

## Author Contributions

HL contributed all the work of the article.

## Funding

This work was supported by the School of Arts, Shandong Management University.

## Conflict of Interest

The author declares that the research was conducted in the absence of any commercial or financial relationships that could be construed as a potential conflict of interest.

## Publisher’s Note

All claims expressed in this article are solely those of the authors and do not necessarily represent those of their affiliated organizations, or those of the publisher, the editors and the reviewers. Any product that may be evaluated in this article, or claim that may be made by its manufacturer, is not guaranteed or endorsed by the publisher.

## References

[ref1] Alaei A. R. Becken S. Stantic B. (2019). Sentiment analysis in tourism: capitalizing on big data. J. Travel Res. 58, 175–191. doi: 10.1177/0047287517747753

[ref2] Aswani R. Kar A. K. Vigneswara I. P. (2018). Detection of spammers in twitter marketing: A hybrid approach using social media analytics and bio inspired computing. Inf. Syst. Front. 20, 515–530. doi: 10.1007/s10796-017-9805-8

[ref3] Barbado R. Araque O. Iglesias C. A. (2019). A framework for fake review detection in online consumer electronics retailers. Inf. Process. Manag. 56, 1234–1244. doi: 10.1016/j.ipm.2019.03.002

[ref4] Bilro R. G. Loureiro S. M. C. Guerreiro J. (2019). Exploring online customer engagement with hospitality products and its relationship with involvement, emotional states, experience and brand advocacy. J. Hosp. Mark. Manag. 28, 147–171. doi: 10.1080/19368623.2018.1506375

[ref5] Cambria E. Poria S. Hussain A. Liu B. (2019). Computational intelligence for affective computing and sentiment analysis [guest editorial]. IEEE Comput. Intell. Mag. 14, 16–17. doi: 10.1109/MCI.2019.2901082

[ref6] Chakraborty K. Bhattacharyya S. Bag R. (2020). A survey of sentiment analysis from social media data. IEEE Trans. Comput. Soc. Syst. 7, 450–464. doi: 10.1109/TCSS.2019.2956957

[ref7] Kaur R. Kautish S. (2019). Multimodal sentiment analysis: A survey and comparison. Int. J. Serv. Sci. Manag. Eng. Technol. 10, 38–58. doi: 10.4018/IJSSMET.2019040103

[ref8] Khalid M. Ashraf I. Mehmood A. Ullah S. Ahmad M. Choi G. S. (2020). GBSVM: Sentiment classification from unstructured reviews using ensemble classifier. Appl. Sci. 10:2788. doi: 10.3390/app10082788

[ref9] Leicht-Deobald U. Busch T. Schank C. Weibel A. Schafheitle S. Wildhaber I.. (2019). The challenges of algorithm-based HR decision-making for personal integrity. J. Bus. Ethics 160, 377–392. doi: 10.1007/s10551-019-04204-w, PMID: 31814653 PMC6868110

[ref10] Li Z. Fan Y. Jiang B. Lei T. Liu W. (2019). A survey on sentiment analysis and opinion mining for social multimedia. Multimed. Tools Appl. 78, 6939–6967. doi: 10.1007/s11042-018-6445-z

[ref11] Lou C. Tan S. S. Chen X. (2019). Investigating consumer engagement with influencer-vs. brand-promoted ads: the roles of source and disclosure. J. Interact. Advert. 19, 169–186. doi: 10.1080/15252019.2019.1667928

[ref12] Lutfi A. A. Permanasari A. E. Fauziati S. (2018). Sentiment analysis in the sales review of Indonesian marketplace by utilizing support vector machine. J. Inf. Syst. Eng. Bus. Intell. 4, 57–64. doi: 10.20473/jisebi.4.1.57-64

[ref13] Polyakova A. G. Loginov M. P. Strelnikov E. V. Usova N. V. (2019). Managerial decision support algorithm based on network analysis and big data. Int. J. Civ. Eng. Technol. 10, 291–300.

[ref14] Rupapara V. Rustam F. Shahzad H. F. Mehmood A. Ashraf I. Choi G. S. (2021). Impact of SMOTE on imbalanced text features for toxic comments classification using RVVC model. IEEE Access 9, 78621–78634. doi: 10.1109/ACCESS.2021.3083638

[ref15] Valle-Cruz D. Fernandez-Cortez V. López-Chau A. Sandoval-Almazán R. (2022). Does twitter affect stock market decisions? Financial sentiment analysis during pandemics: a comparative study of the h1n1 and the covid-19 periods. Cogn. Comput. 14, 372–387. doi: 10.1007/s12559-021-09819-8, PMID: 33520006 PMC7825382

[ref16] Vermeer S. A. M. Araujo T. Bernritter S. F. van Noort G. (2019). Seeing the wood for the trees: How machine learning can help firms in identifying relevant electronic word-of-mouth in social media. Int. J. Res. Mark. 36, 492–508. doi: 10.1016/j.ijresmar.2019.01.010

[ref17] Wang L. Niu J. Yu S. (2019). SentiDiff: combining textual information and sentiment diffusion patterns for twitter sentiment analysis. IEEE Trans. Knowl. Data Eng. 32, 2026–2039. doi: 10.1109/TKDE.2019.2913641

[ref18] Xia H. Yang Y. Pan X. Zhang Z. An W. (2020). Sentiment analysis for online reviews using conditional random fields and support vector machines. Electron. Commer. Res. 20, 343–360. doi: 10.1007/s10660-019-09354-7

[ref19] Yue L. Chen W. Li X. Zuo W. Yin M. (2019). A survey of sentiment analysis in social media. Knowl. Inf. Syst. 60, 617–663. doi: 10.1007/s10115-018-1236-4

